# An observation of two NHS trusts’ use of organisational wellbeing interventions during the COVID-19 pandemic: lessons learnt

**DOI:** 10.1016/j.fhj.2024.100215

**Published:** 2024-11-20

**Authors:** Varun Vijay Kumar, Alexander Deighton, C. Elise Kleyn

**Affiliations:** a Foundation Year 2 Doctor, Midlands Metropolitan University Hospital, Sandwell and West Birmingham NHS trust, Birmingham, UK; b Foundation Year 3 Doctor, Whipps Cross Hospital, Barts Health NHS Trust, London, UK; c The Dermatology Centre, Division of Musculoskeletal & Dermatological Sciences, Faculty of Biology, Medicine & Health (FBMH), The University of Manchester (UoM), Manchester Academic Health Science Centre (MAHSC), Birmingham, UK

**Keywords:** Covid-19, Mental health, Wellbeing

## Abstract

**Background:**

Healthcare workers’ (HW’) mental health during pandemics may be challenged due to the demands of their professional work. There is a need for effective organisational wellbeing strategies so that HW’ mental health is safeguarded. In this article, we sought to examine approaches to implementing wellbeing strategies in hospital trusts, which we hope will improve future workplace planning.

**Methods:**

A pilot study comprising semi-structured interviews with five management staff in two NHS trusts (which employ approximately 5,000 staff each) in north-west England (NWE) were conducted to investigate and compare how COVID-19 impacted HW’ mental health and what wellbeing strategies were implemented.

**Results:**

Lack of guidance and communication, redeployment, fatigue, post-traumatic stress disorder (PTSD) and fear of infection were common stress factors. We identified that both trusts had implemented psychological support, and usage of online resources as well as providing adequate personal protective equipment (PPE). Trust A uniquely implemented psychological screening tools to identify vulnerable HWs, whereas trust B placed an emphasis more on offering practical support to HW.

**Conclusion:**

It is likely that a whole-systems approach to improve organisational culture and reduce stigma around accessing support would be beneficial to HW wellbeing. Further research is required to optimise this support for NHS staff.

## Highlights

• NHS trusts can implement wellbeing strategies to increase access to support.
• Pre-prepared mental health strategies are useful in responding to sudden events.
• Culturally specific strategies are likely to increase engagement from BAME staff.
• Evidence suggests plans in place are inadequate for such events.

## Introduction

It is known that healthcare workers (HW) in the NHS may be subjected to challenging rotas, high-pressure scenarios and the effect of chronic short-staffing placing them at risk of burnout, depression and increased absence due to sickness, the primary cause of which is poor mental health.^1^ It is therefore unsurprising that during pandemics, the mental health of HW may be further challenged due to the demands of their professional work. The implementation of wellbeing strategies by hospitals can subsequently impact HW, enhancing their wellbeing and improving their ability to deliver high-quality patient care. Hospitals can offer support through a variety of initiatives; however, it is important that these are accessible for HW and adequately address their needs.^2^ Despite some areas of good practice within the NHS, where trusts have initiated peer support teams and improved online communication to improve signposting to formal psychological support and offer advice on ways to manage anxiety, overall progress has been insufficient.^3^ The recent (and eventually reversed) cessation of new practitioner health referrals – a primary care service available for NHS staff – exemplifies the lack of a funded and coordinated plan to address these issues comprehensively.^4^ This shortfall is particularly concerning during times of crisis, when existing initiatives are put under immense stress and often prove inadequate in effectively supporting workers’ mental health.^5^^,^^6^

The recent COVID-19 pandemic and the subsequent challenges have further emphasised the need for adequate mental health support programmes. A number of studies in this area have therefore sought to highlight the impact of the pandemic on healthcare staff internationally. Lv *et al* examined the rate of depressive symptoms in Chinese medical staff during the initial wave of the pandemic, demonstrating that of 8,028 staff, one in four exhibited symptoms of insomnia, anxiety or depression.^7^ Further work by Xu *et al* shows these rates to be significantly higher compared with those in pre-pandemic years,^8^ while Yuan *et al* analysed behaviours of 939 medical staff and found poor mental health to increase tobacco and alcohol use, further damaging overall health and quality of life.^9^

Further work outside China supported these findings. A survey of 495 French urology trainees by Abdessater *et al* found that 56% had medium-to-high stress levels during the COVID-19 pandemic. Nevertheless, less than 1% of those sought psychiatric treatment,^10^ a behavioural pattern observed in doctors as a way to maintain ‘high standards of functioning’.^11^ Wu *et al* investigated the factors affecting the wellbeing of 18 nurses working in Wuhan Mental Health Centre during the COVID-19 pandemic. Fear of infection from overcrowded wards was the main factor causing stress, followed by lack of preparation and training for the management of infectious diseases, disruption of the nurses’ personal lives and lack of available childcare during lockdown. Fear of infecting family members was another factor causing stress for HW in Wuhan hospitals, with many not telling their family that they were working in COVID-19 hospitals and others living away from home during the pandemic.^12^ This was further demonstrated with HW in India, where facing stigma, feeling guilty and lack of trust from co-workers further contributed to the poor mental health of HW infected with COVID-19.^13^

Overall, in May 2020 during the COVID-19 pandemic, anxiety, stress and other mental health disorders accounted for 28.3% of all sickness leave in the NHS. The highest sickness absence rate (SAR) in the UK was reported in the north west of England (NWE; 4.9%); with mental health disorders being consistently responsible for sickness absence, accounting for 31.8% of all sickness leave in June 2020.^14^ In addition to staff absences, poor mental health has been shown to manifest in suboptimal outcomes such as errors in the workplace, high staff turnover and poor quality of healthcare delivery.^15^

While a growing body of research exists, further studies are required to explore specific regional variations in wellbeing strategies implemented during the pandemic. In this pilot study, we aim to assess the organisational strategies utilised by two NHS trusts in NWE to improve the wellbeing of their HW between December 2019 and March 2021. In the context of existing clinical leadership theory and systemic promotion of wellbeing in a healthcare setting, we investigated the organisational strategies employed by leaders and managers in UK hospitals to improve the wellbeing of HW in response to the COVID-19 pandemic.

The objectives of the study included:

1. To identify which strategies were used by NHS trusts to improve mental health and wellbeing of HW.
2. To ascertain which risk factors were associated with poor mental health during the COVID-19 pandemic in NHS HW.
3. To examine whether strategies can be designed using limited resources to meet the challenging mental health needs of a diverse background of NHS HW.

## Methodology

### Qualitative data collection and analysis

Ethical approval was granted by the London Southbank University. 1-hour long, semi-structured interviews were conducted via videoconferencing with five participants (clinical managers or directors who had a leadership role within the trust with active involvement in the running or implementation of wellbeing strategies) from two NHS trusts in NWE. The interviews were then transcribed and the data were imported into the NVivo software package to link the concepts contained in the data. The interview questions below (Table 1) were formed in accordance with three frameworks: (1) Health promotion, design and implementation’ Framework by Poland *et al* for implementing healthy behaviours in work settings;^16^ (2) WHO Leadership Framework regarding the need for crisis and financial management;^17^ and (3) NHS Leadership Framework regarding service delivery for leaders.^18^

**Table 1:** List of questions used in semi-structured qualitative interviews.

1.Understanding workplace settings
a) What risk factors for poor mental health during the COVID-19 have been identified to create goals to ensure that the needs of healthcare staff are being responded to?
b) Did the trust have any board level or senior managerial support or champion for mental health wellbeing prior to the pandemic (before March 2020)?
2. Process for implementing strategies to improve wellbeing in workplace settings
a) What organisational strategies had the trust adopted in order to improve healthcare worker wellbeing in response to the pandemic?
b) Would you characterise the trust’s response to mental health due to the pandemic as proactive or reactive?
c) What were the challenges of implementing organisational strategies?
d) Are there any contingency plans to respond to a surge of healthcare workers needing wellbeing support?
3. Ensuring maximum engagement and impact of wellbeing strategies in healthcare settings
a) Can you describe any relationships or partnerships you have developed with other organisations and professional bodies to support your approach?
b) Given the diversity of the workforce, how are specific needs and requirements of staff being addressed?
c) How are budgets for strategies/services evaluated to ensure maximal impact?
d) How important is measuring and assessing the efficacy of strategies for development and improvement?
e) How can IT be used to enhance the performance of organisational strategies to improve worker wellbeing?

The NHS leadership framework outlines a need for healthcare leaders to communicate, collaborate and adapt to the needs of the frontline. The framework incorporates seven domains, but does not offer guidance for leadership strategies during a crisis.^18^

By contrast, the framework ‘Understanding health promotion design and implementation’ by Poland *et al* emphasises the importance of setting a strategy, not merely the influence of the intervention. The framework addresses three steps: (1) understanding settings; (2) changing settings; and (3) knowledge development and knowledge translation for efficient strategy implementation.^16^

The questions developed from these frameworks were then reviewed by a third-party stakeholder with senior management and leadership experience, who provided feedback on their appropriateness and potential bias, which helped avoid eliciting biased or leading responses from participants. Thematic analysis was performed by categorising the qualitative data into meaningful and related categories.

## Results

### Risk factors for poor mental health in the two NHS trusts

The semi-structured interviews identified key risk factors for developing mental health disorders (MHD) (Table 2). These included fear of infection as well as staff concerns over the quality and quantity of PPE. Increased COVID-19-associated workload and redeployment precipitated HW fatigue and MHD.

**Table 2:** Risk factors for poor mental health during the COVID-19 pandemic.

Risk factor	Quotes used by interviewees
Previous mental health disorders	‘Staff who kind of already had a clinical diagnosis of depression, anxiety, PTSD, that was a risk factor’
Poor physical health	‘There was also a large group of staff who were clinically vulnerable from a physical health perspective, which could also impact on their mental health from that anxiety of getting infected’
Uncertainty	‘We didn’t know how long it was going to last’
Personal protective equipment (PPE)	‘Staff raised concerns over the appropriateness of PPE provided by the trust’
Psychological trauma	‘At the start it was mostly about PPE, but then once that had settled down… it moved into their day-to-day experiences, what they were seeing, having to care for these patients who were dying or had potentially died’
Fatigue	‘They had to pick up additional shifts because of other staff shielding or getting sick with COVID and then having to isolate’
Inadequate retraining time	‘There was an anxiety about having to be skilled up in an area they had never worked before’ ‘Redeployment could have been done a bit better… maybe taking into account staff feelings a bit more. It wasn’t the best experience the first time around in terms of the process’
Inadequate guidance and unfair process of redeployment	‘Some staff didn’t really understand why they were being redeployed and others weren’t… then you had the relationship breakdown between colleagues…such as why do I have to go? Why is that person going?… Why am I being put more at risk than them?’ ‘With regards to shielding staff there was the perception… well you’re alright, you’re safe and in your own bubble, but because I’m fit and well I now have to be in the COVID areas’ ‘Since we have gone into work we have had to start building those relationships between colleagues back… perhaps redeployment could’ve been done better, taking into account people’s feelings more’

### Wellbeing strategies

#### Organisational strategies adopted to improve mental health in response to the pandemic

Both trusts adopted diverse strategies to address the risk factors for MHD. (Table 3) Key differences between trusts included:

**Table 3:** Semi-structured interviews identified strategies provided by both trusts to target risk factors for poor mental health.

Type of strategy	Service provided	Targeted risk factors
Psychological support	a) Employee assistance programme - (24/7 access to a counsellor and referral service) (trust A) b) SOS rooms (rest spaces with a mental health first aider/psychologist) (trust A) c) In house psychologist support (trusts A+B) d) Schwartz rounds (trust A) e) Critical incident stress management teams (trust A) f) Mental health first aiders and organisational development staff availability (trusts A+B)	PTSD/moral injury from experiences
Online resources and communication	a) Trust intranet self-care resources (Trust A+B) b) Webinars and forums trust A c) Apps – eg Headspace and Sleepio (trusts A+B)	Lack of communication Unfamiliarity/uncertainty of pandemic
PPE	a) High-quality PPE provided (trusts A+B)	Fear of infection
Practical support initiatives	Providing free bottled water during summer (trust B) Provision of out-of-free hours meals (trust B) Redeployment of admin staff to put on and remove PPE (trust B)	Overworking/fatigue
Psychological screening	a) Resilience workshops to help HW and managers identify signs and symptoms of poor mental health (trust A) b) Health survey – physical and mental health (trust A) c) Shift from annual to quarterly appraisals with managers to discuss wellbeing (trust A)	Unhealthy coping mechanisms PTSD/moral injury from experiences

Trust A reported having critical incident stress management teams and in-house psychologist access. This included a dedicated wellbeing team who co-opted an in-house psychologist to offer staff support. The critical incident stress management team was deployed to provide a conduit for staff feedback from the frontline to the wellbeing and management teams. Trust A also ‘used research from previous pandemics’ (‘swine flu in Toronto’) but due to unforeseen stress factors, their ‘resources weren't enough’ to respond to the pandemic, and ‘required a reactive response’.

Trust B’s wellbeing team comprised managers who committed to wellbeing oversight in addition to their job role. Trust B obtained HW feedback through surveys and provided HW practical support initiatives. Trust B reported a purely reactive response and did not utilise research from previous pandemics.

#### Challenges of strategy implementation and delivery

Participants from both trusts identified a number of challenges to implementation of strategies (Table 4).

**Table 4:** Challenges of strategy implementation and delivery by trust.

Challenges of organisational strategy implementation and delivery	Trust
Lack of psychologist support and resources at the start of pandemic	A
Engagement and awareness of strategies available	A+B
Difficulty recruiting psychologists	A
Unusually low uptake of services by doctors and BAME staff	A
Decision making with minimal guidance	B

### Strategies adopted to enhance staff engagement with wellbeing offering

#### Needs of a diverse workforce

In both trusts, the least engagement with wellbeing strategies was noted with Black, Asian and minority ethnic (BAME) groups. Therefore, trust A and trust B utilised BAME ‘listening events’ and a ‘BAME network’ forum respectively to enable staff to raise concerns. The latter formed part of the ‘governance structure’ of trust B, to facilitate the feedback data being actioned. Trust A also introduced ‘outreach calls’ to ‘check in’ on nursing staff who were on sick leave due to MHD, and onwards referral to the local NHS resilience hub was made if needed. Regular ‘health checks’ were undertaken by HW who consented to being contacted.

#### Evaluation of wellbeing strategies

Both trusts highlighted the importance of measuring the efficacy of strategies implemented. However, trust B reported that due to the ‘fast-paced nature’ of the start of the pandemic, ‘evaluation was not as important’ then. In contrast, trust A submitted quarterly reports on ‘engagement with services, outcomes and feedback’ as part of their service delivery, which they were ‘constantly amending’ to address current need.

## Discussion

The principal analysis highlights strategies adopted by two trusts in NWE to positively influence staff wellbeing during the COVID-19 pandemic. These strategies were designed to mitigate the significant mental health challenges posed by frontline work during the pandemic, where risk factors for mental health difficulties overlapped significantly across both trusts. The main challenges reported included long shifts, limited mental health support, and fear of infection – likely intensified by inadequate PPE, as noted by trust B. Kisley *et al* further emphasised the importance of PPE in meeting HW’ expectations.^19^

Furthermore, the lack of systematic redeployment of HW generated hostility within teams. Frequent guideline changes and rapid retraining proved tiresome for HW, especially if redeployed in an unfamiliar environment. Organisational, as well as team and wellbeing, approaches need to be considered in these emergency settings.^20^

The focus of interventions to support staff in both trusts was individual psychopathology, with minimal exploration of organisational cultural factors. Even though practical support was seen as superior to psychological interventions in trust B, both wellbeing strategies may help improve overworked occupational culture.

Self-care coping mechanisms were emphasised more during the peaks of the pandemic. Similarly, according to Avero *et al*, wellbeing initiatives during the peaks of pandemics should help HW cope with stress and trauma, whereas during recovery phases of pandemics support for processing psychological trauma should be given.^21^ Likewise, trust A hired in-house psychologists only after the peak of the first wave.

Trust A’s screening approach consisted of physical health checks combined with motivational interviewing. It is plausible that identifying common manifestations such as unhealthy eating, smoking and alcohol consumption may be more effective than relying on HW’ self-reporting.^22^ Nevertheless, this system relies on open and honest conversations with HW.

Difficulties in engaging with BAME staff was noted predominantly by trust A. It is known that stigma and negative perceptions towards mental health by be magnified in these individuals and may be ameliorated by delivering culturally sensitive services and utilising existing established communication channels/groups.^23^ In addition, utilising forums to feed into the trust’s formal governance structure allows concerns to be addressed formally.

In both trusts, the least engagement in mental health support was seen among doctors. Despite doctors showing poor self-care, denial and minimising the symptoms of MHD, there are limited wellbeing improvement initiatives tailored for doctors.^11^

In addition to engagement, it is paramount to analyse the efficacy of a particular strategy to ensure effective resource allocation. However, this may be challenging due to the complex nature of mental health and its onset. Trust A regularly submitted quarterly reports analysing engagement, sickness absences due to MHD, and feedback from services. Trust A followed a stepped care model, which is a cost-effective model of care escalation and over time amends services based on data. Interviewees from trust B mentioned that their approach placed less emphasis on collecting data from feedback and engagement from services. Consequently, it may have been more difficult to understand the financial benefits of their strategies and know how to better target funding for future strategies.

These data suggest that when allocating resources at the start of a pandemic, trusts may consider analysing outcomes and limitations of strategies used in previous pandemics and data from specialist organisations such as the British Psychological Society (BPS). Trusts may also benefit from sharing feedback data with other trusts and outsourcing data processing to specialist partner organisations which can reduce staff workloads.

Studies conducted during previous outbreaks have shown that practical support, such as adequate provision of PPE, and voluntary rather than compulsory redeployment, can reduce stress in the healthcare environment.^19^ During the start of COVID-19, the NHS suggested flexible working as the mainstay of mental health support and better working conditions, particularly for HW with dependents.^24^

Current research on how to reduce the adverse mental health impact of COVID-19 emphasises the importance of quickly preparing staff for challenges ahead.^25^ There is evidence that operational training on changes to protocols within an organisation can encourage staff resilience and enhance their capacity to cope during times of high stress.^21^ Virtual video platforms for cognitive behavioural therapy have proven useful for HW due to their flexibility and accessibility without the risk of infection.^26^ ‘Schwartz rounds’, where healthcare workers can share emotionally challenging experiences as a group, have proven to be an effective space for reflection by staff.^27^ Limitations of our study include that it is difficult to pinpoint the direct causal effect of strategies on absenteeism. Additionally, positive outcomes of services may only be seen in the long-term due to the nature of MHD.

Organisation strategies should address risk factors of mental health disorders stemming from systemic issues, such as lack of redeployment training, fatigue from shift-pattern schedules and unclear communication. Trusts may benefit from a more fair, jointly led process of redeployment with HW. Financial board members should ensure adequate COVID-19 wellbeing budgets, in response to continuous outcomes and engagement. Adequate financial and human resource allocations for retraining are required to tackle poor mental health among HW.

Trusts can prioritise eliminating stigma associated with accessing services by improving working culture and team cohesion through campaigns. Careful execution of health-related surveys may help identify HW’ unhealthy coping mechanisms for stress, allowing for earlier detection of MHD. It is important to contextualise the perspective of HW and doctors, particularly of vulnerable population groups such as BAME, and correlate these findings with data collected from service providers.

This study adds to the growing body of work on HW wellbeing, with a focus on the regional context of two NHS trusts. The two NHS trusts in NWE identified similar risk factors for developing MHD and reported similar challenges in implementing wellbeing initiatives. Organisational strategies were dependent on each trust’s needs and outcomes. Screening for psychological issues may highlight required areas of support and may enhance engagement with services, particularly in vulnerable population groups. A definitive study analysing the efficacy of strategies and engagement initiatives with additional quantitative data may indicate the next steps for further improving HW wellbeing. Moreover, a whole-systems leadership approach involving the aforementioned systemic change to organisational culture is required to meet the wellbeing needs of HW, as well as to ensure that we are prepared for challenging or crisis situations that may be encountered.

## Ethical approval and consent to participate

The ethics application was reviewed by London South Bank University and obtained ethics approval (ref: 3905554). All interviewed participants gave written informed consent.

## Funding

This research did not receive any specific grant from funding agencies in the public, commercial, or not-for-profit sectors.

## Data availability statement

The data that support the findings of this study are available from the corresponding author upon reasonable request.

## CRediT authorship contribution statement

**Varun Vijay Kumar:** Conceptualization, Methodology, Investigation, Writing – original draft, Writing – review & editing. **Alexander Deighton:** Writing – original draft, Writing – review & editing. **C. Elise Kleyn:** Methodology, Writing – original draft, Writing – review & editing, Supervision.

## Declaration of competing interest

The authors declare that they have no known competing financial interests or personal relationships that could have appeared to influence the work reported in this paper.
